# Attentional Control Theory in Childhood: Enhanced Attentional Capture by Non-Emotional and Emotional Distractors in Anxiety and Depression

**DOI:** 10.1371/journal.pone.0141535

**Published:** 2015-11-23

**Authors:** Monika A. Waszczuk, Hannah M. Brown, Thalia C. Eley, Kathryn J. Lester

**Affiliations:** 1 King’s College London, MRC Social Genetic & Developmental Psychiatry Centre, Institute of Psychiatry, Psychology and Neuroscience, London, United Kingdom; 2 School of Psychology, University of Sussex, Falmer, Brighton, United Kingdom; Anhui Medical University, CHINA

## Abstract

Attentional control theory (ACT) proposes that anxiety is associated with executive functioning deficits. The theory has been widely investigated in adults. The current study tested whether symptoms of childhood anxiety and depression were associated with experimentally measured attentional control in the context of non-emotional and emotional stimuli. Sixty-one children (mean age = 9.23 years, range = 8.39–10.41) reported their trait anxiety and depression symptoms and completed three visual search tasks. The tasks used a variant of an irrelevant singleton paradigm and measured attentional capture by task-irrelevant non-emotional (color) and emotional (facial expressions) distractors. Significant attentional capture by both non-emotional and emotional distractors was observed, and was significantly correlated with trait anxiety and symptoms of depression. The strength of relationship between attentional capture and the symptoms did not differ significantly for non-emotional and emotional distractors. The results suggest that symptoms of childhood anxiety and depression are associated with poorer attentional control both in the presence of emotional and non-emotional stimuli, supporting ACT in younger populations. This attentional deficit in the context of non-emotional information might be as central to childhood internalizing symptoms as attentional biases often observed on tasks investigating processing of emotional stimuli.

## Introduction

Anxiety and depression are highly prevalent and frequently co-occur across the lifespan [1, 2]. Both disorders have an early age of onset, are very common in young people [1, 3, 4] and reliably predict long-term mental health difficulties [5, 6]. Biases in how individuals attend to, interpret and remember emotional information (particularly negative information) have been implicated in the development and maintenance of internalizing symptoms and disorders [7–10]. These cognitive biases are also targeted by interventions such as cognitive-behavioral therapy (CBT), a recommended first-line treatment for both anxiety and depression [11, 12].

Recent research has begun to investigate attentional processing of non-emotional information in anxious and depressed individuals. Dual-processing theories posit that attentional selection is determined by the competition of two attentional systems: a stimulus-driven, bottom-up system, and a volitional, top-down system [13, 14]. Attentional dysregulation may underlie internalizing disorders [15]. Attentional control theory (ACT) proposes that trait anxiety impairs the efficiency of the volitional system, with bottom-up attentional selection mechanisms overpowering the top-down control system [16, 17]. As a result, anxious individuals are thought to have poorer inhibitory abilities and show more distractibility than non-anxious individuals. ACT is widely supported using non-emotional experimental tasks in adults with anxiety [17, 18], and is also implicated in adults with depression symptoms [19–21]. Furthermore, attentional control serves as a regulatory top-down mechanism moderating emotional attentional biases, with some evidence that biases are only observed in adults with anxiety who show poor attentional control [22, 23]. Thus, general attentional control deficits might in part explain some of the cognitive biases commonly observed on emotional tasks with adults.

There is a growing interest in investigating the relationship between attentional control, internalizing symptoms and cognitive biases in younger populations. Limited empirical evidence indicates an association between poor attentional control and internalizing symptoms in children [24–26]. These results are in line with ACT, suggesting that bottom-up attentional capture overpowers top-down control in children with symptoms of anxiety and depression. Furthermore, high negative emotionality combined with low attentional control was associated with the highest levels of internalizing problems, indicating a moderating effect of attentional control [27–29]. However, many studies used self-reported attentional control, which may be considered a limitation given that questionnaire measures of attentional control generally do not reflect observed behavioral measures of attentional control [23, 30]. Previous research using experimental methods [24, 29, 30] have tended to use relatively complex measures of attentional processes, such as the Stroop task, which are thought to involve various constituents of information-processing, making it difficult to determine which one is associated with internalizing symptoms. Thus, there is a need for a simple experimental attentional control task to study ACT in childhood.

The irrelevant singleton method [31, 32], a relatively pure measure of attentional control, attempts to measure whether the bottom-up system is more dominant than the top-down system during initial attention competition. Participants perform a visual search for a target odd shape in an array of shapes, but on 50% of trials a salient, task-irrelevant color distractor is present. Identification of the unique shape that is unaffected by whether the task-irrelevant distractor is present or not indexes a dominant top-down system. Conversely, the slowing caused by the presence of a distractor indexes the amount of attentional capture via the bottom-up system, providing a direct measure of inhibitory attentional control [33]. Recent electrophysiological studies with adults indicate that reaction times are not confounded by other processes such as decision making or response generation, confirming this task is a specific measure of top-down filtering mechanisms [34, 35]. This measure was significantly correlated with trait anxiety [35, 36] and symptoms of depression and PTSD [37] in adults, supporting ACT.

The current study investigated the relationship between attentional control and symptoms of anxiety and depression in school-aged children. First, attentional control was examined using a standard shape version of the irrelevant singleton task to establish whether experimentally measured attentional control was associated with internalizing symptoms in childhood as indicated in limited studies with largely self-reported attentional control in children. Second, the irrelevant singleton task was adapted to include face stimuli instead of shapes, and participants were asked to search for an odd gender face (rather than an odd shape) in the array. This task (hereafter referred to as the faces-color task) examined whether a task-irrelevant color distractor (an odd colored face) produces attentional capture when performing a visual search amongst face stimuli, and whether this attentional capture is also associated with internalizing symptoms. The third task (referred to as the faces-valence task) also required participants to search for an odd gender face and investigated whether task-irrelevant *emotional* distractors (an odd valenced facial expression; e.g. an angry face amongst an array of neutral faces) elicit attentional capture that is associated with internalizing symptoms. We hypothesized that there would be attentional capture by non-emotional and emotional distractors for visual search among faces, and that greater attentional capture on both face tasks would be associated with symptoms of anxiety and depression. Finally, we compared the magnitude of attentional capture by non-emotional (color) and emotional (face expression) distractors, and their associations with internalizing symptoms. If poor attentional control is as central to childhood anxiety and depression as the attentional biases typically observed on emotional tasks, we expected that attentional capture due to non-emotional and emotional distractors would be similarly associated with internalizing symptoms.

## Method

### Participants

Ethical approval was granted by the Psychiatry, Nursing and Midwifery Research Ethics Subcommittee of King's College London (ref no: PNM/12/13-54). Participants were recruited from a primary school in London, UK. Written consent was obtained from parents and verbal assent from children. Sixty-one children (mean age = 9.23 years, SD = .57, range: 8.39–10.41) participated (34% response rate) of which 52.46% were male, 95.08% right-handed and 90.20% classified as Caucasian; which is comparable to the UK general population [38].

### Stimuli and Materials

#### Questionnaires

Trait anxiety was assessed using the Trait Anxiety Inventory for Children (STAIC-T) [39]. Children indicated using keyboard buttons how often (hardly ever, sometimes, often) the 20 questionnaire items were true for them. Depression symptoms were assessed using the Short Mood and Feelings Questionnaire (SMFQ) [40], a 13-item self-report measure assessing whether (not true, sometimes, true) symptoms of depression occurred in the previous two weeks. Higher scores indicate higher trait anxiety and depression symptoms, respectively. The psychometric properties of both measures are very good [40–42] and the internal consistencies in the current study were high (α = .85 and .81 for STAIC-T and SMFQ respectively). Anxiety and depression were highly correlated (*r* = .70, *p* < .001). Additionally, in order to capture overall emotional problems, a composite internalizing score was formed with unit-weighted z-scores of anxiety and depression measures.

#### Attentional Tasks

**Fig 1:**
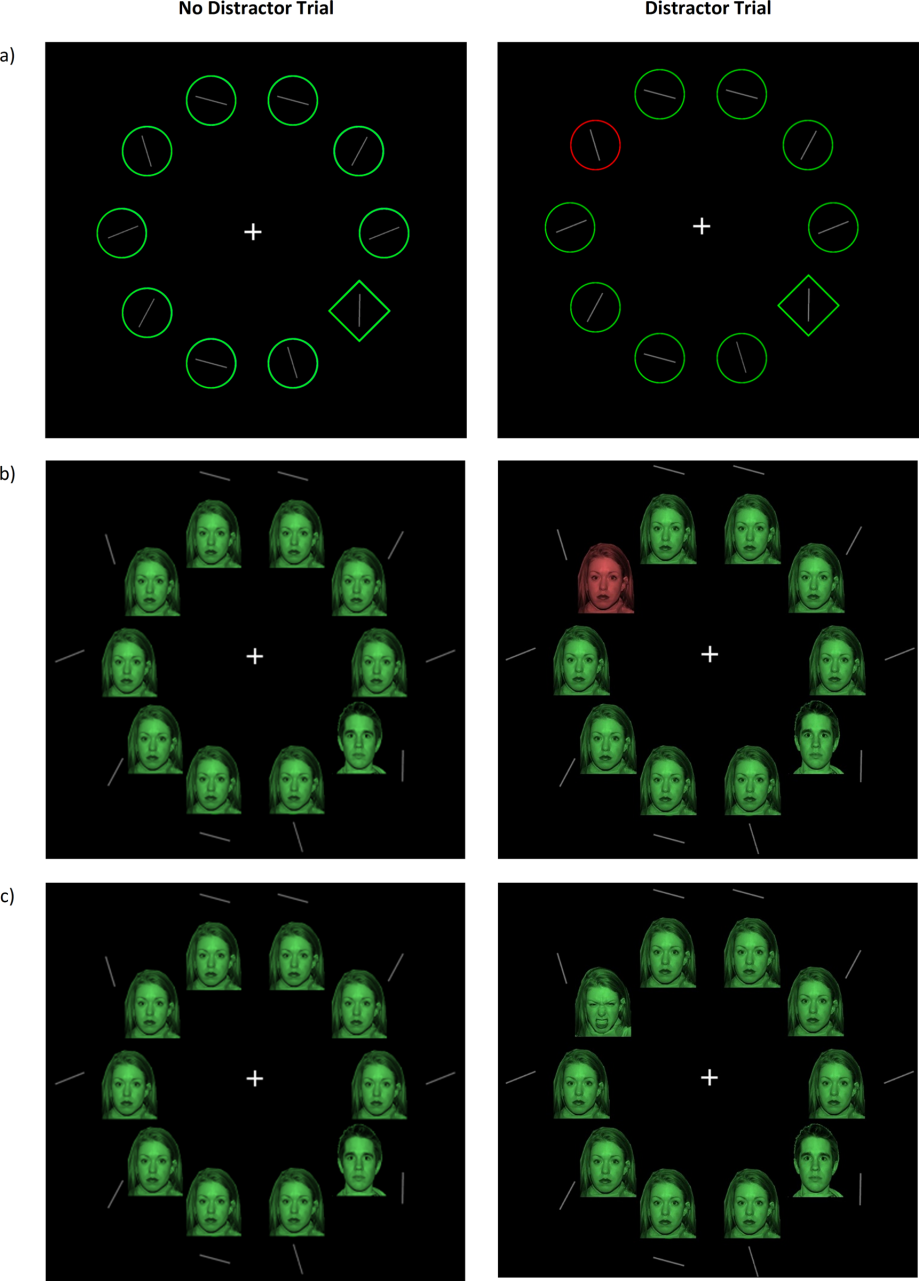
Example of no-distractor and distractor trials for each of the three tasks used in the study: a) Shapes task, b) Faces-color task, c) Faces-valence task. In shapes task example, on both trials a diamond is a target shape and participants are required to indicate by button press that the line inside is vertical. On a distractor trial one of the circles in the array is of a task-irrelevant distractor of opposite color (red).In faces-color and faces-valence tasks examples, a male face is a target face and participants are required to indicate by button press that the line next to it is vertical. In faces-color task example on a distractor trial one of the female faces in the array is of a task-irrelevant distractor of opposite color (red). In faces-valence task example on a distractor trial one of the female faces in the array is of a task-irrelevant distractor of opposite valence (an angry facial expression).

Shapes task. The shapes task was adapted from a study in adults [36]. Ten shape outline stimuli (diamonds and circles) were presented in random order spaced in an imaginary circle around a fixation point on a black background (*Fig 1A*). The shapes always contained a grey line segment presented centrally. The line segments were oriented vertically, horizontally, or tilted 22.5° to the right or left of the horizontal or vertical plane. The search array comprised nine identical shapes and one odd target shape (e.g. a diamond amongst circles for 50% of trials and a circle amongst diamonds on the other 50%). The odd target shape contained vertical lines on 50% of the trials, and horizontal lines on the remaining trials (randomly assigned), while the rest of the array shapes contained the randomly assigned tilted line segments. Half of all trials contained no distractor; all 10 shapes were the same color (either green or red). The distractor trials were identical, but one of the nine non-target shapes was selected at random to appear in the opposite color to the other shapes. Participants were required to find the odd shape in the array and identify whether the line inside was horizontal or vertical.

Faces-color task. The aim of the faces-color task was to establish whether there is attentional capture by an irrelevant color singleton amongst an array of face stimuli. The shape stimuli were substituted with two faces selected from the NimStim face set [43]. The faces-color task is a novel adaptation of the shapes task with two modifications. Participants were required to find the odd gender face in the array, and identify whether the line next to the odd gender face was horizontal or vertical. On 50% of trials a single male face target appeared amongst 9 identical female faces and vice versa for the remaining 50% of trials. The faces were shaded green or red, and on distractor trials (50% of all trials) one face appeared in the opposite color. The line segments were presented next to the face in an outer circle in order to not interfere with the face image (*Fig 1B*).

Trials were randomly presented in two blocks, one with all emotionally neutral faces and one where all faces had an angry facial expression. Both blocks were used so that faces-color and faces-valence task (see below) contained identical no-distractor trials and were counterbalanced on facial expressions used, allowing for direct comparison between the two tasks. The identity of the male and female face remained constant across blocks.

Faces-valence task. The aim of the faces-valence task was to establish whether there is attentional capture by an irrelevant emotional singleton amongst an array of face stimuli, and whether the magnitude of this ‘emotional’ capture differs from the extent of attentional capture by a color distractor amongst an array of face stimuli on the faces-color task. In this task, participants were again required to identify whether the line next to the odd gender face was horizontal or vertical. However, instead of the presence of a color-face distractor, there was a facial expression distractor. On the no-distractor trials (50% of all the trials), all 10 faces shared the same facial expression (either neutral or angry). The distractor trials were identical, but one of the nine non-target faces was randomly selected to appear in the opposite facial expression to the other faces. For example, if nine faces had a neutral expression (eight identical female faces and one target male face, all the same color), the distractor face had an angry expression (an angry female face of the same identity and color as the remaining female faces). Thus, half the trials consisted of an angry distractor amongst neutral faces, and half consisted of a neutral distractor amongst angry faces (*Fig 1C*).

Trials were presented in two blocks in randomized order; one with all green faces and one with all red faces. Both blocks were used so that faces-color and faces-valence tasks contained identical no-distractor trials and were counterbalanced on colors used, allowing for direct comparison between the two tasks. As with the faces-color task, the identity of the male and female face remained constant across blocks.

### Procedure

All tasks were programmed in E-Prime 2.0 (Psychology Software Tools, Pittsburgh, PA). Children were supervised by a researcher during a 1-hour session undertaken individually in a quiet classroom. Instructions and questionnaire items were read aloud to ensure comprehension. The children and the school each received a voucher for participating.

The questionnaires were completed first in a randomized order, followed by the three experimental tasks. For comparison with other published studies, the shapes task was always completed first, to prevent introducing potential carry over effects of the face task. The four blocks of face tasks (two for the faces-color task and two for the faces-valence task) were presented next, in a randomized order.

The images were displayed on a laptop (13.3” display with 1366 x 768 screen resolution) and each image was size 130×178 pixels. Children were instructed to press a button on a keyboard (L for ‘horizontal’, A for ‘vertical’, both labelled clearly) to indicate whether the line segment belonging to the target shape or face was horizontally or vertically oriented. In accordance with previous research using the irrelevant singleton task [36], they were instructed to ignore any color/facial expression information and focus solely on finding the odd shape/gender face. Reaction time (RT) was recorded. Each task consisted of 160 trials, with complete counterbalancing of the 10 target locations × 2 target line orientations × 2 target shape colors/face colors/face expressions × 2 target shape/gender face × 2 distractor conditions. On distractor trials, the distractor location was random. Across the three tasks participants completed 480 trials in total, with breaks in-between blocks and tasks.

Each trial began with a fixation point presented for a randomly selected time ranging from 600-1200ms in 100ms increments, followed by a search array displayed until the participant responded. Auditory feedback (a short beep sound) was given for incorrect responses. The next trial began after a 1-second black frame.

#### Data preparation and analysis strategy

For comparison with published studies, where possible the analyses followed a previously used approach [36]. Task accuracy on all tasks was very high (Shapes task: μ = 96%, SD = 3%; Faces-color task: μ = 96%, SD = 6%; Faces-valence task: μ = 97%, SD = 5%). No participant was excluded based on overall poor accuracy (<60%). Two participants did not receive auditory feedback during the task and were excluded from analyses. One participant terminated the study after the shapes task. Participants were removed from analyses when their overall mean RT was 2 SD above or below the overall sample mean RT in order to exclude extremely slow or fast participants. The resulting sample size was 56 for shapes task and 57 for faces-color and faces-valence tasks. Trials below 250ms or 2.5 SD above each participant’s mean RT per trial type were removed to exclude extremely slow and fast trials (7.5% of trials for shapes task, 5% for face tasks). Then each participant’s mean RT on correct trials was calculated separately for distractor and no distractor trials.

A repeated measures analysis of variance (ANOVA), including two factors (task: shapes, faces-color and faces-valence; trial type: distractor and no distractor) was conducted on mean RTs to establish baseline experimental effects and to compare the three tasks. Distractor cost was calculated for RT on each task (distractor minus no distractor). Bivariate correlations were conducted between the distractor cost on each task and trait anxiety, depression and the composite internalizing score. The magnitude of correlation coefficients was compared using a z-test procedure [44]. In order to assess the unique association between RT distractor cost and trait anxiety/symptoms of depression, partial correlations were conducted, controlling for the depression/anxiety scores respectively.

The analyses were repeated for accuracy. Accuracy was very high across all three tasks and showed little variance. There were no significant differences in accuracy between task or trial type. The accuracy distractor cost (distractor trials accuracy minus no-distractor trials accuracy) on each of the three tasks was not significantly associated with trait anxiety, depression symptoms, or the composite internalizing score.

## Results

The mean trait anxiety score was 33.18 (SD = 6.80; range = 22–49) and the mean depression symptoms score was 4.34 (SD = 3.75, range = 0–19). The scores were comparable to community norms: trait anxiety normative scores for males were μ = 36.30 (SD = 6.80) and females μ = 38.10 (SD = 6.06) (Spielberger, 1973); depression symptoms normative score was μ = 4.68 (SD = 4.66) (Angold et al., 1995). There were no significant age or sex differences.

Preliminary analyses compared within-task distractor RT cost (distractor trials minus no-distractor trials) for the two blocks comprising each face task. For the faces-color task, there was no significant difference in the distractor cost when the array comprised all neutral faces compared to all angry faces (*t*(50) = 0.43, *p* = .67). Likewise, there was no significant difference in distractor cost on the faces-valence task when the array comprised all red faces versus all green faces (*t*(54) = .48, *p* = .63) or when the distractor was an angry face relative to a neutral face (*t*(56) = 0.46, *p* = .65). There was also no difference in RT on no-distractor trials when the array comprised of all neutral vs all angry faces (*t*(50) = 1.23, *p* = .23 for faces-color task, *t*(54) = .71, *p* = .48 for faces-valence task). Thus, RTs and distractor cost were calculated collapsing across the two blocks within each face task. For mean RT comparisons between female vs male face arrays, see *S1 Table*.

**Fig 2:**
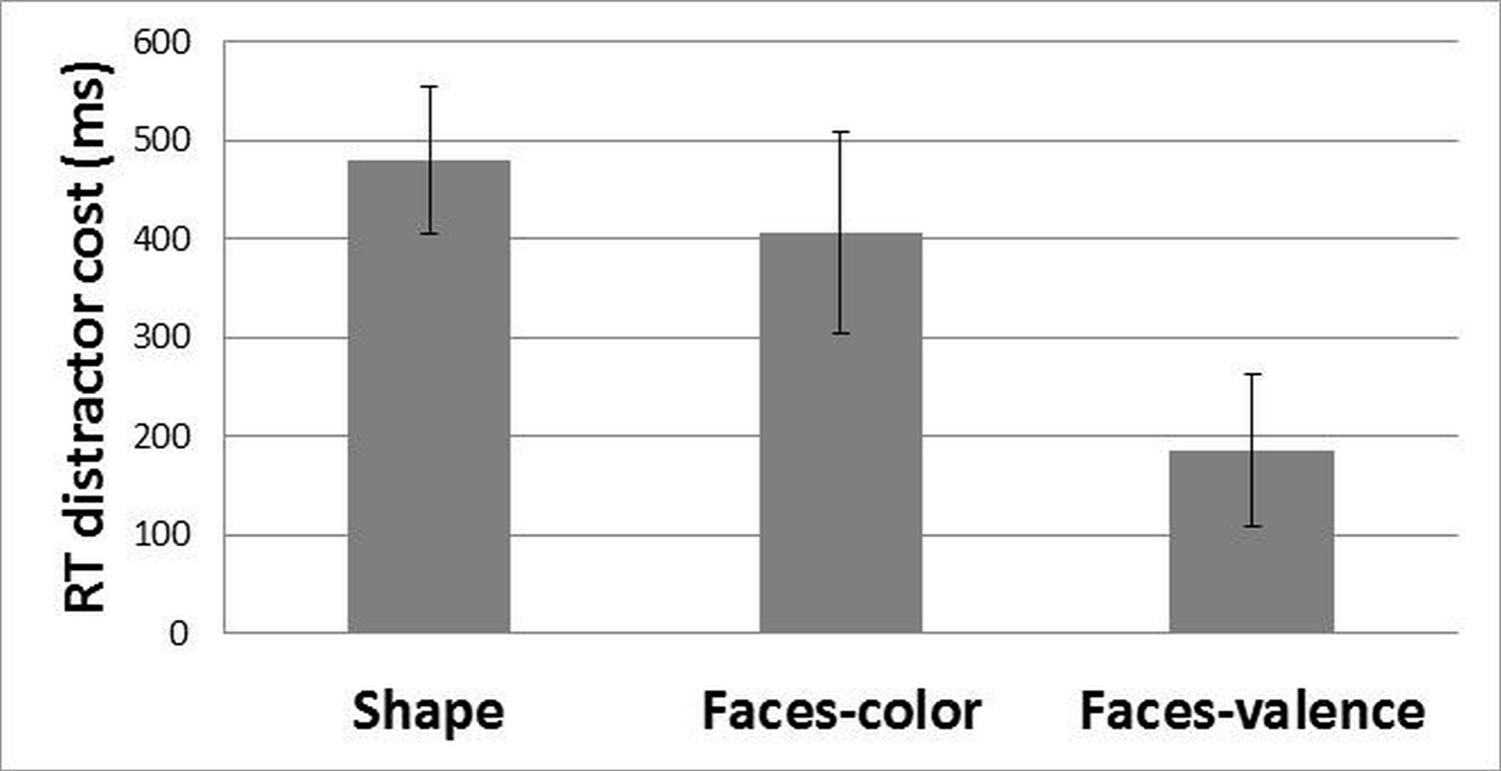
RT distractor cost for the shapes, faces-color and faces-valence tasks. Error bars indicate 95% confidence intervals. Non-overlapping confidence intervals indicate significant difference between the mean scores.

Participants were significantly slower on distractor compared to no distractor trials (*F* = (1,53) = 164.40, *p* < .001, *η*_*p*_^*2*^ = .76; 2914.87ms vs. 2547.19ms) with this effect observed for all three tasks (*Table 1*). Overall, participants were significantly slower to perform the faces-color task (3097.73ms) compared to the faces-valence task (2956.86ms, *p* = .003) and slower on both face tasks relative to the shape task (2138.51ms, *p’s* < .001; *F* = (1.72, 91.04, Huynh-Feldt correction) = 185.68, *p* < .001, *η*_*p*_^*2*^ = .78). Finally, there was a significant task × trial type interaction (*F*(2,106) = 16.97, *p* < .001, *η*_*p*_^*2*^ = .24). To tease apart this interaction, the distractor costs were entered into a repeated measures ANOVA with Task as the within participant variable. The effect of Task was significant (*F* = (1.83, 93.48, Huynh-Feldt correction) = 11.17, *p* < .001, *η*_*p*_^*2*^ = .18) with a smaller distractor cost for the faces-valence task compared to the faces-color (*p* = .01) and shape task (*p* < .001) but no significant difference between the faces-color and shape task (*p* = 1.00), suggesting that color was more distracting than valence (*Fig 2*).

**Table 1:** RT on distractor and no-distractor trials, and RT distractor cost for each task, and each block within faces tasks.

*Task*	*N*	*RT Distractor (ms)*Mean (SD)	*RT No distractor (ms)*Mean (SD)	*Distractor Cost (ms)*Mean (SD)
**Shape (all trials)**	**56**	**2391.24 (537.34)**	**1911.25 (392.20)**	**479.99 (285.89)**
**Faces-color (all trials)**	**57**	**3349.43 (701.23)**	**2944.04 (693.59)**	**405.39 (392.86)**
*Neutral face array*, *red/green distractor*	*55*	3378.12 (709.50)	2926.22 (584.92)	451.90 (501.36)
*Angry face array*, *red/green distractor*	*53*	3267.47 (735.95)	2863.13 (736.42)	404.34 (456.45)
**Faces-valence (all trials)** ^**a**^	**57**	**3075.44 (634.44)**	**2889.84 (584.78)**	**185.60 (299.46)**
*Green face array*, *angry/neutral distractor*	*57*	3142.82 (726.40)	2942.46 (673.47)	200.36 (372.46)
*Red face array*, *angry/neutral distractor*	*55*	3035.44 (659.99)	2842.45 (586.80)	192.99 (367.69)

^a^ Faces-valence distractor trials can also be divided into:

Angry face array, neutral face distractor cost = 170.75 (448.80) (relative to mean faces-valence no distractor RT)

Neutral face array, angry face distractor cost = 200.45 (313.08) (relative to mean faces-valence no distractor RT)

Trait anxiety and the composite internalizing score were significantly correlated with the distractor cost for each task (*Table 2*). Depression was significantly correlated with distractor cost for the shape and faces-valence task, but while in the expected direction, it did not reach statistical significance for the faces-color task. The strength of association between distractor cost and trait anxiety, depression and internalizing score was not significantly stronger when the distractor was emotionally valenced compared to a non-emotional color distractor (irrespective of face or shape task, *z’s* = -1.28-.48, *p’s*>.05, see *Table 2*footnote for all comparisons). Within the faces-valence task, distractor cost due to a neutral distractor was correlated with symptoms of anxiety (r = .34) and depression (r = .27) to the same extent as distractor cost due to an angry distractor (r = .33 for anxiety, r = .31 for depression, all *p* < .05).

**Table 2:** The correlation between RT distractor cost for each task, and trait anxiety, depression symptoms and composite internalizing score.

Task distractor cost	Trait Anxiety	Depression Symptoms	Composite Internalizing
Shapes	.27*	.30*	.31*
Faces-color	.26*	.16	.23
Faces-valence	.40*	.38*	.43*

* is *p* < .05

The correlations were not significantly different: Correlations with trait anxiety: Faces-color vs. Faces-valence: *r* = .26 vs .40, *z* = -.84, *p* = .20; Shapes vs. Faces-valence: *r* = .27 vs .40, *z* = -.78, *p* = .22; Faces-color vs. Shapes: *r* = .26 vs .27, *z* = -.06, *p* = .47; Correlations with depression: Faces-color vs. Faces-valence: *r* = .16 vs .38, *z* = -1.28, *p* = .10; Shapes vs. Faces-valence: *r* = .30 vs .38, *z* = -.48, *p* = .32; Faces-color vs. Shapes: *r* = .16 vs .30, *z* = -.88, *p* = .19; Correlations with composite internalizing: Faces-color vs. Faces-valence: *r* = .23 vs .43, *z* = -1.20, *p* = .12; Shapes vs. Faces-valence: *r* = .31 vs .43, *z* = -.73, *p* = .23; Faces-color vs. Shapes: *r* = .23 vs .32, *z* = -.51, *p* = .30

Symptoms of anxiety and depression were highly correlated (*r* = .70, *p* < .001) making it problematic to confidently decompose their unique contribution to distractor costs. Partial correlations revealed that neither anxiety nor depression uniquely predicted distractor costs for each task.

The overall mean RT on each task was not significantly correlated with symptoms of anxiety/depression (.15-.26, *p* = .06-.26), except for a significant correlation between anxiety symptoms and an overall RT on faces-valence task (r = .27, *p* = .04). Looking at the correlations separately for no-distractor and distractor trials, the correlations between symptoms of anxiety/depression and mean RT on no-distractor trials (which indicate baseline performance) were all non-significant (r = .06-.20, *p* = .14-.63). The correlations with mean RT on distractor trials were generally significant (r = .21-.35, *p* = .01-.12). The results suggest that the presence of the distractor produces the RT differences as a function of trait anxiety and depression.

## Discussion

### Results summary

The current study was the first to investigate ACT in middle childhood using an irrelevant singleton paradigm. In line with our hypotheses, poorer attentional control, measured as attentional capture by task-irrelevant non-emotional and emotional distractors, was significantly correlated with trait anxiety and symptoms of depression, directly supporting ACT in a school-age sample. The strength of relationship between attentional capture and internalizing symptoms did not differ significantly for non-emotional and emotional distractors. This finding suggests that heightened symptoms of anxiety and depression in childhood are not only associated with attentional processing biases in the presence of emotional information but also appear to be associated with a general attentional deficit for non-emotional stimuli.

### Attentional control theory in childhood

Our finding that trait anxiety and symptoms of depression are associated with enhanced attentional capture by a distractor is consistent with our hypothesis, and with previous research demonstrating poorer attentional control in anxious adults using this paradigm [35–37] and with evidence from child populations using other paradigms [24–26]. However, the current study extends previous findings in several important and novel ways. First, the irrelevant singleton task is a more precise measure of attention than self-report measures or the more complex tasks employed by previous adult and child studies, such as the Stroop task, which are likely to involve multiple cognitive processes. The current method, used here for the first time in young participants, allows direct measurement of the extent to which bottom-up attentional capture dominated top-down control during the initial allocation of attention. The simplicity of the current task (demonstrated by high accuracy on all task variants) is especially important when testing child participants, whose executive functions are not fully developed [45] and for whom any additional processes such as memory, language or response selection might interfere with performance to greater degree than in adults. Of note, we observed a significantly smaller distractor cost using emotional face expressions relative to non-emotional color distractors (shape or face), which is consistent with evidence that color is more salient than facial expression [46].

Second, ACT is less often investigated in relation to depression than anxiety, especially in children. Our results suggest that poor attentional control is not unique to anxiety as originally proposed by ACT; instead it is associated with a general internalizing symptomatology. This is in line with transdiagnostic views of anxiety and depression, which argue that many cognitive mechanisms are shared between internalizing disorders [47–50].

### Attentional control and attentional biases

Third, this study is the first to extend the irrelevant singleton paradigm to study attentional control in the context of emotional distractors. In line with our hypothesis, we found that attentional capture due to emotional and non-emotional distractors was similarly associated with internalizing symptoms, indicating impaired attentional control of non-emotional and emotional processing in anxiety and depression. This tentatively suggests that attentional control deficits might in part account for some of the cognitive biases observed in anxious and depressed participants on tasks using emotional stimuli. That is, attentional biases on emotional tasks might reflect poor attentional control rather than solely selective processing of emotional (e.g. threat) stimuli. This is consistent with previous studies, which found that anxious individuals showed bias towards threat only when their self-reported attentional control was low [22, 27, 28, 51]. It suggests that dominant bottom-up attentional system might be a risk factor for maladaptive information-processing in anxiety and depression, in line with dual-processing models [15]. Furthermore, no difference in distractor cost on the faces-valence task was observed for angry vs. neutral face distractors, and these distractor costs were comparably associated with symptoms of anxiety and depression, suggesting that attentional capture was not specific to negatively valenced distractors.

Although observed in a population-based sample and in need of direct study in samples with clinically elevated symptoms, the association between poor attentional control and elevated symptoms of childhood anxiety and depression indicates attentional training as a potential treatment and prevention target for internalizing problems. Preliminary evidence suggests that attentional control training might be successful at reducing internalizing symptoms [52, 53]; although the effectiveness of cognitive training approaches is currently debated [54, 55]. Importantly for targeting childhood mental health problems, younger participants seem to benefit more from cognitive training than adults [54], possibly reflecting greater neural and behavioral plasticity earlier in development. Finally, future research should explore whether established therapies such as CBT, as well as novel approaches such as attentional-bias modification training (ABM) [56], improve overall attentional control. This could provide novel insight into the mechanisms of action underpinning these therapeutic processes [15].

### Limitations

The use of a sensitive behavioral task and its novel adaptation to investigate the role of ACT in the context of emotional stimuli are considerable strengths of the current study. However, there are a number of limitations. First, the sample size was underpowered to investigate sex differences or detect small effects. In addition, we did not detect statistically significant differences in the magnitudes of the correlations between internalizing symptoms and the distractor costs due to non-emotional and emotional distractors. Effect sizes were similar for each of these comparisons. Overall, the results should be replicated in a larger sample. Second, although the relatively narrow age-range allows closer understanding of a specific developmental stage, it limits the generalizability of the results to other ages. ACT needs to be investigated longitudinally to clarify age-related changes in attentional control and its associations with internalizing symptoms across development. Third, in order to replicate the adult study as closely as possible, the shapes task was always completed before the face tasks, thus the carryover effects of the shapes task on the face tasks are not accounted for. However, the faces-color and faces-valence task were fully counterbalanced and therefore performance on these tasks can be compared. Finally, other reasons for individual differences in distractor cost, such as sex differences and hyperactivity symptoms, should be explored in larger, adequately powered samples. Future studies should also utilize eye-tracking techniques to disentangle whether increased vigilance or difficulty disengaging from the distractors underpinned attentional capture.

### Conclusions

In conclusion, the current study applied and extended the irrelevant singleton method in order to investigate the relationship between attentional control for both non-emotional and emotional stimuli and symptoms of anxiety and depression in school-aged children. The results demonstrated that childhood symptoms of anxiety and depression are associated with poorer attentional control, supporting ACT in a school-age population. This general attentional deficit might characterize children with elevated symptoms of anxiety and depression and may work alongside or even underpin attentional biases, which are often observed on tasks investigating selective processing of emotional stimuli in this population. Future research should investigate whether attentional control may play a role in the etiology and maintenance of cognitive biases and explore the therapeutic potential of attentional training approaches for anxiety and depression.

## Supporting Information

S1 Dataset The dataset containing variables used in the study. (SAV)

S1 Table Mean RT on face tasks where array comprised of all female vs all male faces, presented separately for no-distractor and distractor trials. On all female arrays, the target was the odd male face. On all male arrays, the target was the odd female face. The distractor was the same sex as the array but either had opposite colour (faces-colour task) or opposite valence (faces-valence task). Repeated measures ANOVA indicated that there were significant differences between mean RT: Face colour: *F*(2.58, 128.41, Huynh-Feldt correction) = 48.26, *p* < .001, η_p_^2^ = .49. There was no significant difference between males and females on no-distractor trials (*p* = .52), which suggests that it doesn’t matter whether the target is male or female. However on distractor trials performance on the ‘male’ array was slower than on the ‘female’ array (*p* = .02). This suggests that the male colour distractor was more distracting than the female colour distractor. Both female and male colour distractors produced slower RTs as compared to no-distractor trials. Face valence: *F*(2.29, 123.71, Huynh-Feldt correction) = 21.05, *p* < .001, η_p_^2^ = .28. There was no significant difference between males and females on no-distractor trials (*p* = .38), suggesting that it doesn’t matter whether the target is male or female. However on distractor trials performance on the ‘male’ array was slower than on the ‘female’ array (*p* = .00). This suggests that the male valence distractor was more distracting than the female valence distractor. In this task we also found that RTs on female valence distractor trials were not significantly slower than RTs on no-distractor female trials (*p* = .53).
(DOCX)
