# Supplementary material for: Attentional Control Theory in Childhood: Enhanced Attentional Capture by Non-Emotional and Emotional Distractors in Anxiety and Depression
Source: PLoS One. 2015 Nov 23;10(11):e0141535. doi: 10.1371/journal.pone.0141535 (PMC4658135; doi:10.1371/journal.pone.0141535)
Supplement: S1 Table — On all female arrays, the target was the odd male face. On all male arrays, the target was the odd female face. The distractor was the same sex as the array but either had opposite colour (faces-colour task) or opposite valence (faces-valence task). Repeated measures ANOVA indicated that there were significant differences between mean RT: Face colour: F(2.58, 128.41, Huynh-Feldt correction) = 48.26, p < .001, ηp 2 = .49. There was no significant difference between males and females on no-distractor trials (p = .52), which suggests that it doesn’t matter whether the target is male or female. However on distractor trials performance on the ‘male’ array was slower than on the ‘female’ array (p = .02). This suggests that the male colour distractor was more distracting than the female colour distractor. Both female and male colour distractors produced slower RTs as compared to no-distractor trials. Face valence: F(2.29, 123.71, Huynh-Feldt correction) = 21.05, p < .001, ηp 2 = .28. There was no significant difference between males and females on no-distractor trials (p = .38), suggesting that it doesn’t matter whether the target is male or female. However on distractor trials performance on the ‘male’ array was slower than on the ‘female’ array (p = .00). This suggests that the male valence distractor was more distracting than the female valence distractor. In this task we also found that RTs on female valence distractor trials were not significantly slower than RTs on no-distractor female trials (p = .53). (DOCX) [file pone.0141535.s002.docx]

**S1 Table** – Mean RT on face tasks where array comprised of all female vs all male faces, presented separately for no-distractor and distractor trials.

|  | No-distractor trials | Distractor trials |
| --- | --- | --- |
| Face colour |  |  |
| RT on array where all faces are female | 2804.95 | 3171.86 |
| RT on array where all faces are male | 2871.59 | 3409.86 |
| Face valence | | |
| RT on array where all faces are female | 2847.10 | 2932.84 |
| RT on array where all faces are male | 2947.88 | 3277.71 |

Notes:

On all female arrays, the target was the odd male face. On all male arrays, the target was the odd female face. The distractor was the same sex as the array but either had opposite colour (faces-colour task) or opposite valence (faces-valence task).

Repeated measures ANOVA indicated that there were significant differences between mean RT:

Face colour: F=(2.58, 128.41, Huynh-Feldt correction)=48.26, p<.001, η_p_^2^=.49. There was no significant difference between males and females on no-distractor trials (p=.52), which suggests that it doesn’t matter whether the target is male or female. However on distractor trials performance on the ‘male’ array was slower than on the ‘female’ array (p=.02). This suggests that the male colour distractor was more distracting than the female colour distractor. Both female and male colour distractors produced slower RTs as compared to no-distractor trials.

Face valence: F=(2.29, 123.71, Huynh-Feldt correction)=21.05, p<.001, η_p_^2^=.28. There was no significant difference between males and females on no-distractor trials (p=.38) , suggesting that it doesn’t matter whether the target is male or female. However on distractor trials performance on the ‘male’ array was slower than on the ‘female’ array (p=.00). This suggests that the male valence distractor was more distracting than the female valence distractor. In this task we also found that RTs on female valence distractor trials were had not significantly slower than RTs on no-distractor female trials (p=.53)
